# Robust Inclusion Complex of Topotecan Comprised within a Rhodamine-Labeled β-Cyclodextrin: Competing Proton and Energy Transfer Processes

**DOI:** 10.3390/pharmaceutics15061620

**Published:** 2023-05-30

**Authors:** Maria Rosaria Di Nunzio, Abderrazzak Douhal

**Affiliations:** Departamento de Química Física, Facultad de Ciencias Ambientales y Bioquímica and INAMOL, Universidad de Castilla-La Mancha, Av. Carlos III, s/n, 45071 Toledo, Spain; mrosaria.dinunzio@uclm.es

**Keywords:** anticancer drugs, fluorescent cyclodextrins, host–guest systems, spectroscopy techniques, drug delivery

## Abstract

Monitoring the biological fate of medicaments within the environments of cancer cells is an important challenge which is nowadays the object of intensive studies. In this regard, rhodamine-based supramolecular systems are one of the most suitable probes used in drug delivery thanks to their high emission quantum yield and sensitivity to the environment which helps to track the medicament in real time. In this work, we used steady-state and time-resolved spectroscopy techniques to investigate the dynamics of the anticancer drug, topotecan (TPT), in water (pH ~6.2) in the presence of a rhodamine-labeled methylated β-cyclodextrin (RB-RM-βCD). A stable complex of 1:1 stoichiometry is formed with a K_eq_ value of ~4 × 10^4^ M^−1^ at room temperature. The fluorescence signal of the caged TPT is reduced due to: (1) the CD confinement effect; and (2) a Förster resonance energy transfer (FRET) process from the trapped drug to the RB-RM-βCD occurring in ~43 ps with 40% efficiency. These findings provide additional knowledge about the spectroscopic and photodynamic interactions between drugs and fluorescent functionalized CDs, and may lead to the design of new fluorescent CD-based host–guest nanosystems with efficient FRET to be used in bioimaging for drug delivery monitoring.

## 1. Introduction

**Scheme 1:**
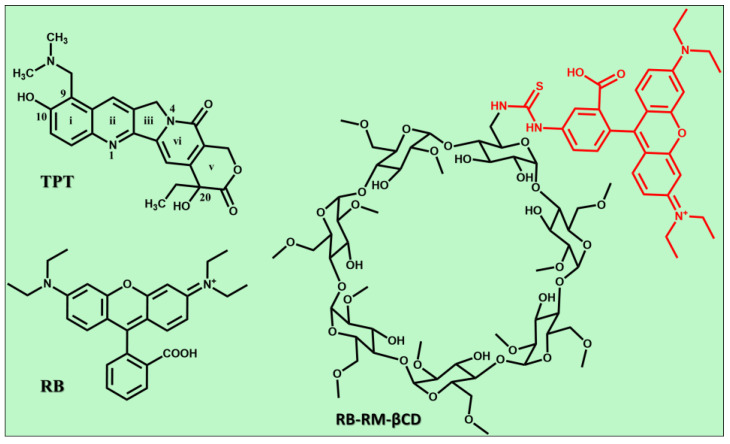
Molecular structures of topotecan (TPT) in its lactone form, rhodamine B (RB), and RB-RM-βCD.

The anticancer drug topotecan (TPT, Scheme 1) is a camptothecin (CPT)-analogue that has been proven to exert topoisomerase I (Top1) inhibition. Cancer cells are usually killed by damaging the ribonucleic acid (RNA) or deoxyribonucleic acid (DNA) necessary for cell division [1]. TPT inhibits cell division by preventing DNA synthesis and hindering the topoisomerase activity [2]. TPT shows higher solubility in water and lower cytotoxicity in human tissues compared to the case of its parent compound CPT [3]. Moreover, both intravenous and oral TPT administration have been permitted for the treatment of several cancers [4,5,6,7,8,9,10]. One of the major drawbacks of TPT is that it undergoes the reversible hydrolysis of the v-lactone ring depending on the surrounding pH [11]. At a lower pH (≤6), the lactone closed-ring form predominates over the carboxylate open-ring one, whose concentration increases, in turn, at higher pHs (Scheme S1) [12]. The lactone and carboxylate forms show a different pharmacological activity as the anticancer activity of the lactone form is greater than that of the carboxylate one [13]. Clinical tests revealed that, in plasma, the TPT-lactone concentration rapidly decreases with a mean half-life of 3.4 h, and lactone hydrolysis and renal excretion constitute the principal ways of elimination of the drug [14]. This issue can be sidestepped by the use of nanocarriers which protect TPT from hydrolysis until the active drug reaches the acidic pH levels of the endosome (pH = 5.5–6.0) or lysosome (pH = 5.4–5.0) organelles [15]. Recently, a variety of organic and inorganic nanocarriers such as liposomes, nanoparticles, and metal–organic frameworks have been proposed as TPT nanotherapeutics [16,17,18,19,20,21,22,23,24,25,26,27,28].

Among the organic nanocarriers, cyclodextrins (CDs) and CD-based nanoparticles have been employed as efficient TPT-hosting systems which lead to improved TPT solubility/stability and pH-controlled drug release behavior [29,30,31,32,33]. Furthermore, in vivo test cellular investigations have demonstrated a significant increase in cellular uptake and the cancer cell death of TPT:CD-based nanoparticle complexes with respect to the free drug [31,33]

Both native and substituted CDs such as 2-hydroxypropyl-βCD (HP-βCD), sulfobutylether-βCD (SBE-βCD), and randomly methylated βCD (RM-βCD) are used in a wide variety of practical applications including catalysis, chromatography, bio-nanotechnology, pharmacy, and medicine [34,35]. The use of CD complexation in drug delivery has been broadly reviewed and supported by numerous in vitro and in vivo studies [36,37,38,39,40,41,42,43,44,45].

Nevertheless, the use of spectroscopy represents a significant tool to unravel the ground- and excited-state behavior of supramolecular systems. To this end, intensive studies of CD inclusion complexes such as drug-delivery nano-carriers has been performed by steady-state and time-resolved spectroscopic techniques [30,46,47,48,49,50,51,52,53,54,55,56,57,58,59]. These investigations were dedicated to obtain deep insights into the effects of confinement on the photophysics and photochemistry of the molecular guests such as: the formation of specific and non-specific interactions, emission intensity growth/decrease, excimer/exciplex formation, photocleavage, charge- and proton transfer (CT and PT) reactions, energy transfer (ET), and *cis*–*trans* photoisomerization for improving both drug design and delivery [60].

Fluorophore-labeled CDs are among the most suitable systems for detecting the encapsulation of guest molecules since their inclusion results in guest-induced spectroscopic modifications which depend on the degree of the host–guest interaction [61,62]. Fluorophores are directly attached to the CD window, thus giving birth to sophisticated supramolecular architectures to be used as labeled molecular carriers in cell cultures or biofilms in order to follow their uptake (ability to cross biological barriers) and intracellular localization and spatial distribution [58,59,63,64,65,66,67,68]. Xanthene derivatives such as fluorescein, eosin, and rhodamine are among the most applied fluorophores in the synthesis of emissive CDs. In particular, thanks to their high absorption coefficients, remarkable emission quantum yields, and pH insensitivity, rhodamine dyes are widely used as molecular probes in biotechnological applications such as fluorescence microscopy, flow cytometry, fluorescence correlation spectroscopy, and Enzyme-Linked ImmunoSorbent Assay (ELISA) [69,70,71,72,73,74,75,76,77,78,79,80,81,82,83].

As a fluorescence- and distance-based mechanism, the FRET phenomenon plays a key role in exploring the interaction between a nanomedicine and its biological environment. The aim is to have a real control over the intracellular and in vivo drug “biofate”, which is considerably related to the clinical therapeutic effect of the medicament. The topic of FRET measurements in cells treated with rhodamine-based supramolecular systems has previously been studied and is still of great interest nowadays [84,85,86,87]. FRET generates fluorescence signals that are susceptible to molecular conformation, association, and separation at a scale of 1–10 nm [88]. One important aspect of a FRET-based sensing technique is that it does not directly produce redox-active ions that could lead to photodamage or other undesirable processes.

In a previous report, the spectroscopy and dynamics of TPT were investigated in the aqueous buffered solutions of three different βCDs, including the native and methylated ones, respectively, heptakis(2,6-di-O-methyl)-βCD (DM-βCD) and heptakis(2,3,6-tri-O-methyl)-βCD (TM-βCD) [30]. We observed that the CD environment influences the deactivation pathways of caged TPT, modifying the rate of the non-radiative processes upon its encapsulation. Additionally, proton nuclear magnetic resonance ([1]HNMR) experiments and semi-empirical (PM3) calculations have suggested that the docking of TPT with the CDs occurs across the quinoline moiety.

Here, using steady-state and time-resolved spectroscopy techniques, we explored the interaction between TPT and a fluorescent CD, 6-deoxy-6-[(5/6)-rhodaminylthioureido]-RM-βCD (RB-RM-βCD, Scheme 1), in aqueous solutions at a near neutral pH (~6.2). A robust 1:1 complex formation was confirmed by the high value of the complex stability constant (K_eq_ = 4.0 ± 0.9 × 10^4^ and 3.4 ± 0.6 × 10^4^ M^−1^ from two independent experiments), which is reminiscent of those previously found for TPT in the presence of DM-βCD (K_eq_ = 2.4 ± 0.5 × 10^4^ M^−1^) and TM-βCD (K_eq_ = 3.7 ± 0.8 × 10^4^ M^−1^) [30]. The increased hydrophobic character of the hosting system turns the ground-state equilibrium of caged TPT towards the neutral form of the drug in accordance with our previous results. In addition, the TPT complexation with RB-RM-βCD induces the efficient quenching of the fluorescence intensity compared with the cases of DM-βCD and TM-βCD, thus suggesting a FRET process between the confined drug and RB molecules covalently bonded to the CD cage. The occurrence of a FRET is further endorsed by any time-resolved experiments, which shed light on the photodynamics of the TPT:RB-RM-βCD complex. By resolving the FRET equations, we estimated a TPT-to-RB ET efficiency of 40%. These results contribute to enhancing our knowledge about the ground- and excited-state behaviors of drugs complexed with fluorescent CDs. In addition, the interesting photodynamical aspects of this material make it a potential candidate to be used in bioimaging to track intracellular TPT release by monitoring the variation in the RB emission.

## 2. Materials and Methods

TPT ((S)-(+)-topotecan hydrochloride) (Merck, Schnelldorf, Germany, ≥98%), RB-RM-βCD (6-deoxy-6-[(5/6)-rhodaminylthioureido] randomly methylated-βCD, average degree of substitution for RB = 0.5–1.0; average degree of substitution for Me groups = 9.0–13.0) (CycloLab, Budapest, Hungary) was used. The buffer solution (pH = 7.3) was prepared using doubly distilled water following a standard protocol. The TPT and RB-RM-βCD solutions were prepared within the concentration ranges of 5.50–28.24 and 0.11–110 µM, respectively. The steady-state UV–visible absorption and fluorescence spectroscopic experiments were performed using JASCO V-670 and FluoroMax-4 (Jobin-Yvone, Longjumeau, France) spectrophotometers, respectively. The ps-ns time-resolved emission measurements were recorded with a time-correlated single-photon counting (TCSPC) system [89]. The samples were excited by 40 ps-pulsed (~1 mW, 40 MHz repetition rate) diode-lasers (PicoQuant, Berlin, Germany) centered at 371 nm. The instrumental response function (IRF) was ~70 ps. The collected decays were deconvoluted and fitted to multi-exponential global functions by applying the FLUOFIT package (PicoQuant). Both the fit quality and number of exponentials were meticulously evaluated based on the reduced χ^2^ values (which were always < 1.2) and the distributions of the residuals. The multi-exponential fits for the studied complexed systems originate from the existence of different emitters in solutions, as we demonstrated. We tried to obtain an accurate fit using a model involving 2 or 3 exponential functions. However, we obtained larger χ^2^ values (>1.2) and a poor distribution of the residuals, indicating the need for more exponentials to fit the data. All the experiments were performed at room temperature (20 °C).

## 3. Results

### 3.1. Steady-State Study

#### 3.1.1. UV–Vis Absorption Spectra

**Figure 1:**
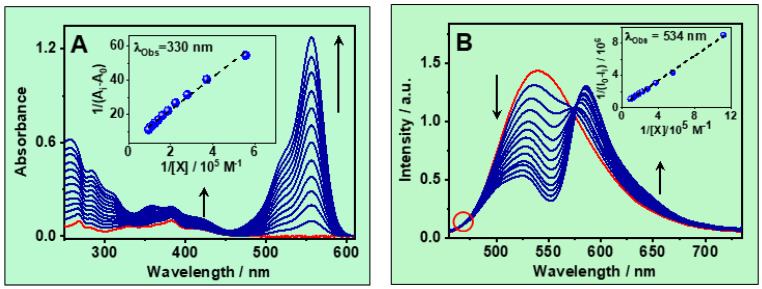
(**A**) Absorption and (**B**) emission spectra (excitation wavelength = 371 nm) of TPT 5.5 µM in water solutions (pH ~6.2) without (red line) and after (blue lines) the addition of different aliquots of RB-RM-βCD. The blue spectra are corrected for the fraction of light absorbed by TPT. The red circle in (**B**) indicates the position of an iso-emissive point at ~470 nm. Insets: the Benesi–Hildebrand plots from absorption (**A**) and fluorescence (**B**) titration data of TPT with RB-RM-βCD (denoted as X in the insets) are observed at (**A**) 330 and (**B**) 534 nm. The obtained K_eq_ values from the two methods are: (**A**) 4.0 ± 0.9 × 10^4^ and (**B**) 3.4 ± 0.6 × 10^4^ M^−1^.

To date, it has been reported that the presence of multifunctional groups makes TPT go through several equilibria between different structures depending on the pH of the solution [90]. The pK_a_ values which were empirically calculated are pK_a1_ < 0.8 and pK_a2_ ~3.6, corresponding to the protonation of N1 and N4 sites, respectively; and pK_a3_ = 6.5 and pK_a4_ = 10.7, relative to the deprotonation of 10-hydroxyl and protonated 9-dimethylaminomethylene groups, respectively (Scheme 1). Based on these data, we proposed three structures of the TPT in equilibrium in slightly acidic water solutions (pH = 6.24): enol (E, $\lambda_{abs}^{max}$ = 374 nm), cation (C, $\lambda_{abs}^{max}$ = 382 nm), and zwitterion (Z, $\lambda_{abs}^{max}$ = 409 nm), where the E form can either have a closed or open configuration (Scheme S2) [91]. In particular, open E is ascribed to an E networking with water molecules through intermolecular H-bonds (iHBs). Under these conditions, anion (A) is not involved in the ground-state equilibrium, but it is generated in the excited state. Water-dissolved CPTs are well known to undergo the hydrolysis of the lactone v-ring, yielding a relatively inactive and more toxic carboxylate form (pK_a_ of carboxylic group ~6.5) [12,92]. The hydrolysis efficiency increases at low proton concentrations [12,92]. Moreover, the presence of a hydroxyl group at the 10-position as in 10-hydroxycamptothecin helps to stabilize the lactone increasing its half-life from ~17 min to ~22 min in phosphate-buffered saline (PBS) solutions at 37 °C [93]. On the basis of these considerations, we decided to study the interaction of TPT with the RB-RM-βCD host under acidic physiological conditions (pH ~6.2), in order to reduce the percentage of TPT-carboxylate in equilibrium with TPT-lactone as much as possible; the former was calculated to be 30% for TPT in an aqueous solution at pH 6.24 [91]. As a last consideration, for 10-hydroxycamptothecin derivatives in water and water/MeOH mixtures, it has been experimentally proven that v-ring hydrolysis does not basically modify the ground- or excited-state behaviors of these systems [94]. Hence, we cannot neglect the co-existence of TPT-lactone and TPT-carboxylate forms (open E, C, Z, and photoproduced A) in water at a near neutral pH. Nevertheless, they should have very similar spectroscopic (absorption and emission spectra) behaviors and excited-state dynamics. Figure 1 shows the absorption and emission spectra of TPT 5.5 µM in water solutions at pH ~6.2 without and after the addition of increasing aliquots of RB-RM-βCD ([RB-RM-βCD] from 0 to 10.7 µM).

**Figure 2:**
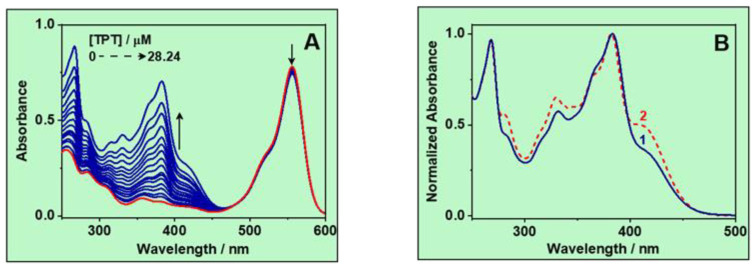

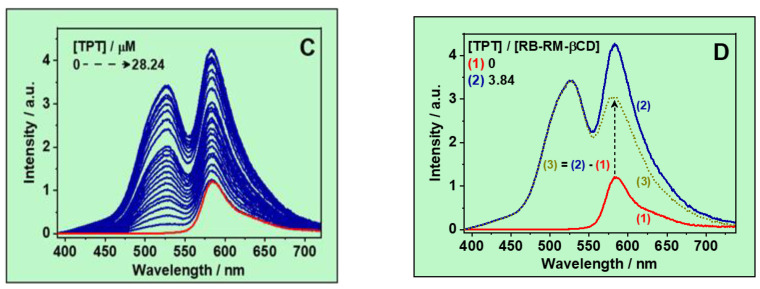
(**A**) Absorption spectra of RB-RM-βCD 7.35 µM in water solutions (pH ~6.2) without (red line) and after (blue lines) the addition of different aliquots of TPT. (**B**) Normalized (to the maximum intensity) absorption spectra of TPT:RB-RM-βCD (1, solid line) and pristine TPT (2, dashed line) in aqueous solutions. Contribution from RB-RM-βCD to the total spectrum of the complex (spectrum 1) is subtracted in order to only represent the absorbance values related to the drug. (**C**) Emission spectra of RB-RM-βCD 7.35 µM in water solutions (pH ~6.2) without (red line) and after (blue lines) the addition of different aliquots of TPT. (**D**) Comparison between the spectrum of the complex TPT:RB-RM-βCD (2) and that of free RB-RM-βCD (1) in aqueous solutions. Contribution from RB-RM-βCD (1) to the total spectrum of the complex (2) is subtracted to obtain the spectrum (3). The excitation wavelength is at 371 nm.

It has been reported that, in the presence of three different βCDs, including native and methylated ones (DM-βCD and TM-βCD, respectively), a decrease in the Z population of TPT with a concomitant increase in the E population of TPT can be observed upon increasing the amount of CD [30]. The high value of K_eq_ (3.7 ± 0.8 × 10^4^ M^−1^) obtained for the TPT:TM-βCD complex indicates the formation of a more favorable interaction between the guest (the E form of TPT) and the host thanks to its larger hydrophobic character with respect to βCD (K_eq_ = 0.88 ± 0.09 × 10^4^ M^−1^) and DM-βCD (K_eq_ = 2.4 ± 0.5 × 10^4^ M^−1^). In our case, it was difficult to distinguish the spectral evolution of TPT in the presence of RB-RM-βCD due to the strong absorption of RB in the whole investigated spectral range (210–610 nm). Therefore, in order to verify the spectroscopical changes occurring for the caged TPT, the latter was added in increasing amounts to a starting aqueous solution of RB-RM-βCD ~7 µM up to reach a [guest]/[host] (guest = TPT; host = RB-RM-βCD) ratio of ~4 (Figure 2A).

High guest concentrations were used with the aim of shifting the equilibrium towards the products (in this case, the TPT:RB-RM-βCD complex). Figure 2B shows a comparison between the absorption spectrum of the TPT:RB-RM-βCD complex (1, after subtracting the RB-RM-βCD contribution) and that of the pristine TPT (2). In the presence of RB-RM-βCD, we can appreciate a decrease in the 409/281 and 331 nm absorption bands, corresponding to the Z and C forms of the drug, respectively, in favor of a larger amount of E, in accordance with our previous results. The absorption intensity maxima of TPT:RB-RM-βCD agree with those found for the TPT:DM-βCD and TPT:TM-βCD complexes (Figure S1). Based on the absorption and ^1^HNMR results, in a previous work, we suggested that, in the presence of βCD and its methylated βCDs, a portion of the drug (iii-, iv-, and v-rings) is still interacting with the neighboring water molecules [30] so we can draw similar conclusions for the case of TPT:RB-RM-βCD. Therefore, the hydrolysis of the v-ring of either free or trapped TPT must be considered under these experimental conditions. However, if the caged TPT-carboxylate and caged TPT-lactone coexist, they should display very similar spectroscopic (absorption and emission spectra) and dynamical properties.

#### 3.1.2. Emission Spectra

Figure 1B shows the emission spectra of TPT in water at pH~6.2 upon excitation at 371 nm (close to the absorption maximum of caged E) and in the presence of increasing quantities (up to 10.7 µM) of RB-RM-βCD. The emission band at 580 nm comes from the RB moiety attached to the RM-βCD. Since RB-RM-βCD also absorbs at this excitation wavelength (Figure S2), the spectra recorded after adding the host to the solution are corrected for the fraction of light solely absorbed by TPT. At a near neutral pH, the emission of TPT mainly comes from Z* ($\lambda_{em}^{max}$ = 540 nm, $\Delta {\overset{-}{\nu }}_{ST(Z^{*})}$ ~ 7700 cm^−1^), while the blue-emitting open E* ($\lambda_{em}^{max}$ = 421 nm, $\Delta {\overset{-}{\nu }}_{ST(E^{*})}$ ~ 2400 cm^−1^) is not appreciable [91]. The A* species ($\lambda_{em}^{max}$ = 556 nm, $\Delta {\overset{-}{\nu }}_{ST(A^{*})}$ ~ 8200 cm^−1^), which makes almost no contribution in the ground-state, is generated in the excited-state by the deprotonation of the photo-excited open E* [91]. Nevertheless, its emission band is not visible because it is hidden by the one coming from Z*. The fluorescence from C* ($\lambda_{em}^{max}$ = 455 nm) has only been observed in ps-time-resolved emission spectra (TRES) [91]. Both the position and shape of the TPT emission band have shown no change in the aqueous solutions of βCD, DM-βCD, and TM-βCD [30]. The absence of the confinement effect in the emission behavior was explained in terms of a partial exposure of a caged TPT to the water molecules outside the CD cavity. Furthermore, the low emission intensities observed for caged E* were justified by a very efficient conversion of E* into Z*, even within the hosting cavity [30]. In line with these preceding results, an iso-emissive point at ~470 nm is perceptible from the emission spectra of TPT:RB-RM-βCD (Figure 1B), only suggesting a modest increase in the emission intensity of caged E*. However, in this case, the TPT emission band drastically changes both in shape (FWHM reduction from ~3200 to ~2400 cm^−1^) and position ($\lambda_{em}^{max}$ shifts from 540 to 525 nm) when RB-RM-βCD is gradually added to the starting water-dissolved TPT (Figure 1B). In the presence of βCD and its methylated analogues, we observed a general decrease in the emission efficiency of TPT, with Φ_F_ values of ~0.2, for βCD and DM-βCD, and ~0.1, for TM-βCD [30]. The lowering of Φ_F_ of the caged TPT with respect to that measured in the THF (0.38), a solvent with a polarity comparable to that of the CD interior, was explained in terms of the presence of an encapsulated, short-living A* which does not exist in THF. Now, if we compare the I/I_0_ ratio (I_0_ and I are the emission intensities at 540 nm for the free drug in the absence and presence of CD, respectively) calculated for TPT:DM-βCD (I/I_0_ = 0.77) and TPT:TM-βCD (I/I_0_ = 0.56) with that found in the case of TPT:RB-RM-βCD (I/I_0_ = 0.29), we see that the complexation with RB-RM-βCD provokes the maximum TPT fluorescence quenching among the analyzed systems. Based on a very good spectral overlap between the emission and absorption spectra of TPT and RB, respectively (Figure S3), we can rationally ascribe the extra-emission reduction detected in TPT-RB-RM-βCD to a FRET process between TPT (donor, D) and RB (acceptor, A).

The solutions used in the absorption experiments were also used in the fluorescence experiments (Figure 2C). Figure 2D shows a comparison between the emission spectrum of the TPT:RB-RM-βCD complex at the highest [guest]/[host] value (~4). It is clear from the spectra that the emission of the RB moiety is enhanced in the presence of TPT, thus reinforcing the suggestion of a TPT-to-RB ET process. It has been reported for TPT:βCD, TPT:DM-βCD, and TPT:TM-βCD complexes that at least two ground-state E forms of TPT co-exist within the CD cavity: (1) a red-shifted absorbing species assigned to a closed, non-interacting E producing Z* upon direct excitation; and (2) a blue-shifted species corresponding to an open E probably interacting with the water molecules at the primary (small) gate of CD [30]. The latter undergoes an excited-state deprotonation to give A*. Therefore, we suggest a similar behavior for the TPT:RB-RM-βCD compound studied herein. Ps-time-resolved experiments will give further information on the aforementioned emission data and will clarify the involvement of species in the FRET process (*vide infra*).

#### 3.1.3. Determination of the Complex Stability Constant (K_eq_)

To obtain the complex stability constant (K_eq_) for the involved equilibria between TPT and the hosting RB-RM-βCD, both the absorption and emission spectra were treated with the Benesi–Hildebrand (BH) model, whose details are given in the Supporting Information. The inset of Figure 1A shows the variation in the inverse of the absorption intensity difference (A_i_-A_0_) at 330 nm, where A_0_ and A_i_ are the absorption values of TPT in the absence and presence of CD, respectively, vs. 1/[RB-RM-βCD]. We chose this observation wavelength because, at these regions, the contribution of RB to the total absorption spectrum is minimum (Figure S2), so we can appreciate the absorbance changes in the complex. On the other side, the inset of Figure 1B shows the variation in the inverse of the emission intensity difference (I_0_-I_i_) at 534 nm, where I_0_ and I_i_ are the emission values of TPT free and upon addition of CD, respectively, vs. 1/[RB-RM-βCD]. The data were fitted supposing a 1:1 stoichiometry, which was confirmed by high R^2^ values (≥0.99). Two very similar K_eq_ values were obtained: 4.0 ± 0.9 × 10^4^ and 3.4 ± 0.6 × 10^4^ M^−1^ from the absorption and emission datasets, respectively. These two values resemble those found for the complexation of the drug with the methylated βCDs, DM-βCD (K_eq_ = 2.4 ± 0.5 × 10^4^ M^−1^), and TM-βCD (K_eq_ = 3.7 ± 0.8 × 10^4^ M^−1^),^30^ demonstrating the efficient formation of a stable complex. The interaction of TPT with βCD and hydroxypropylated-βCD (HP-βCD) was investigated in acidic (pH = 3.5 and 6) buffered solutions containing 18% ethanol [29]. These complexes did not show great stability, and the binding constants at pH 6 are 13 ± 1 and 14 ± 1 M^−1^ for TPT:βCD and TPT:HP-βCD, respectively. Nevertheless, more recently, water-soluble negatively charged CD derivatives such as heptakis-[6-deoxy-6-(3-sulfanylpropanoic acid)]-βCD (H1) and heptakis-[6-deoxy-6-(2-sulfanylacetic acid)]-βCD (H2) showed significant high binding abilities towards TPT of up to (1.5 ± 0.2) × 10^5^ M^−1^.^32^ The interest in these systems consisted in their pH-controlled release behaviors: the anticancer drug could be efficiently encapsulated in the CD cavity at pH 7.2, like that of serum, and then efficiently released at pH 5.7, which is the endosomal pH value of a cancer cell.

As we have previously shown by the use of semi-empirical PM3 calculations, the docking of TPT with pristine and methylated βCDs occurs through the quinoline moiety, which presents the highest degree of penetration within the cavity [30]. Based on these results, we suggest that, also for the TPT:RB-RM-βCD complex studied here, the most favorable encapsulation of the drug is across its quinoline part.

### 3.2. Ps-Time-Resolved Emission Study

**Figure 3:**
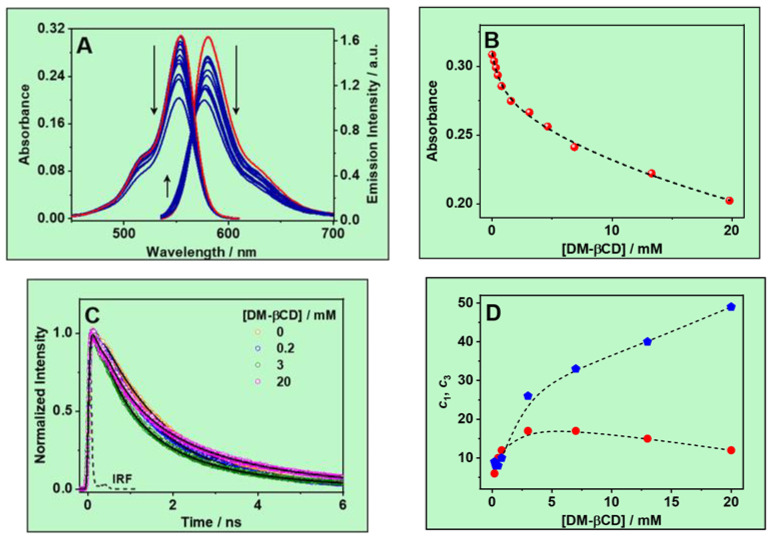
(**A**) Absorption and emission spectra of RB 2.9 µM in water solutions (pH ~6.2) without (red line) and after (blue lines) the addition of DM-βCD at different concentrations (from 0.2 to 20 mM). The blue spectra are corrected for the dilution effect. (**B**) Absorbance variation of RB in water at pH ~6.2 with a DM-βCD concentration observed at 554 nm. The dashed line is from the best fit assuming the formation of 1:1 and 1:2 complexes using Equation (S7). (**C**) Normalized (to the maximum of intensity) ps-emission decays of RB 2.9 µM in water solutions (pH ~6.2) without and after the addition of DM-βCD at different concentrations (0.2, 3, and 20 mM). The excitation and observation wavelengths are 371 and 560 nm, respectively. The solid lines are from the best fit of the experimental data. IRF is the instrumental response function. (**D**) Changes in the contributions obtained for the decay components *τ*_1_ (*c*_1_) and *τ*_3_ (*c*_3_) as functions of DM-βCD concentration. The dashed lines in (**D**) are just to guide the eyes.

Emission Lifetimes. To explore the photophysics of the RB-RM-βCD host, we first studied the interaction between RB and DM-βCD in water with increasing amounts of the latter (up to 20 mM). Steady-state experiments (Figure 3A) revealed that, at lower DM-βCD concentrations (from 0.2 to 0.8 mM), the dye interacts with the host by forming a supramolecular complex showing a reduction in both its absorption and emission spectra but without changing the position of their intensity maxima.

**Scheme 2:**
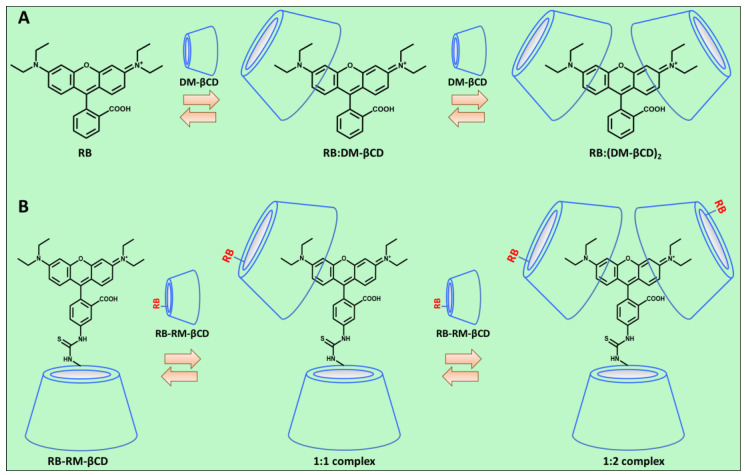
Possible interactions between (**A**) RB and DM-βCDs and (**B**) among RB-RM-βCDs in water solutions.

Nevertheless, at higher DM-βCD concentrations (from 3 to 20 mM), the absorption/emission reduction is also accompanied by a weak hypsochromic shift in the intensity maxima (Figure S4). According to previous reports [95], we assign these changes to the formation of 1:1 and 1:2 stoichiometry complexes between RB and DM-βCD, namely RB:DM-βCD and RB:(DM-βCD)_2_, respectively (Scheme 2A). Figure S5 compares the fit to two complexes (1:1 and 1:2) with the fit to only one complex (1:1), indicating the better quality obtained with the first one, especially at low concentrations of DM-βCD. The absorbance reduction and blue-shift are ascribed to a partial loss of planarity of the molecular structure of the dye, with a consequent decrease in its π-conjugation. To obtain the binding constants for these complexes, we used Equation (S7), and the best fit gave K_1_ = 1.1 ± 0.5 × 10^3^ M^−1^ and K_2_ = 20 ± 2 M^−1^ (Figure 3B). The formation of the 1:1 and 1:2 complexes between RB and DM-βCD was also supported by time-resolved ps-experiments (Figure 3C,D and Figure S6 and Table 1 and Table S1). The free RB decays in a mono-exponential fashion with a lifetime of 1.67 ns. In the presence of DM-βCD, apart from the component related to the free dye in the solution (*τ*_2_), we observed shorter (*τ*_1_ = 560–600 ps) and longer (*τ*_3_ = 3.3–3.9 ns) time constants.

The contribution of the *τ*_1_ (*c*_1_) component shows a maximum value (17) at (DM-βCD) = 3–7 mM, while that of *τ*_3_ (*c*_3_), being rather small (8–10) at host concentrations between 0.2 and 0.8 mM, starts to rapidly increase at [βCD] = 3 mM until reaching a maximum value (49) at [DM-βCD] = 20 mM (Figure 3C and Figure S6B).

Considering these results, we assign the lifetimes *τ*_1_ = 560–600 ps and *τ*_3_ = 3.3–3.9 ns to the 1:1 and 1:2 complexes, respectively. This assignment is further confirmed by the very close similarity of *τ*_1_ and *τ*_3_ to the lifetimes recorded for the 1:1 (610 ps) and 1:2 (3.36 ns) complexes between RB and βCD in a phosphate buffer at pH = 6 [95].

**Figure 4:**
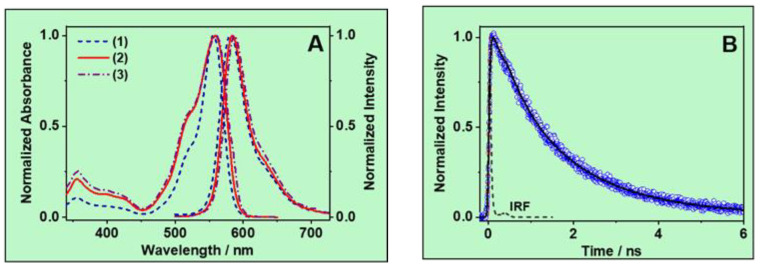
(**A**) Absorption and emission spectra of differently concentrated solutions (1.1 × 10^−6^ M, (1); 1.1 × 10^−5^ M, (2); 1.1 × 10^−4^ M, (3)) of RB-RM-βCD in PBS at pH = 7.3. (**B**) Normalized (to the maximum of intensity) ps-emission decays of RB-RM-βCD in PBS solutions (pH = 7.3) at two different concentrations (1.1 × 10^−4^ and 1.1 × 10^−7^ M) and observed at 640 nm. The excitation wavelength is at 371 nm. The solid lines are from the best fit of the experimental data. IRF is the instrumental response function.

Secondly, we investigated the ground- and excited-state properties of different concentrated (from 1.1 × 10^−7^ to 1.1 × 10^−4^ M) solutions of RB-RM-βCD in PBS at pH = 7.3, as shown in Figure 4.

Figure S7 shows the emission decays of such samples, gated throughout the whole RB-RM-βCD emission wavelength range (565–670 nm). Table S2 collects the corresponding time constants (τ_i_), normalized pre-exponential factors (a_i_), and contributions (c_i_) obtained from the global multi-exponential fits of the emission decays. At all the used CD concentrations, the analysis gives three components with lifetimes of: *τ*_1_ = 580–590 ps, *τ*_2_ = 1.6–1.7 ns, and *τ*_3_ = 3.3–3.5 ns. The intermediate time constant, *τ*_2_, is assigned to the emission the from RB attached to the primary CD gate due to its similarity to that of the free dye in the water solutions (*vide supra*). *τ*_2_ displays the highest contribution, which is ~80% over the whole observation range and at all the used concentrations. The shortest and longest lifetimes, *τ*_1_ and *τ*_3_, have contributions of 6–11 and 10–12%, respectively, which show only small fluctuations within the observation wavelength and do not appreciably change with CD concentrations. As the *τ*_1_ and *τ*_3_ values are very similar to those found for RB in the presence of DM-βCD, we ascribe them to the lifetimes of 1:1 and 1:2 complexes, respectively, between the attached RB and one or two RM-βCDs (Scheme 2B). Notice that, due to the restriction imposed by the aliphatic arm bonding the two moieties in the RM-β-CD, the RB moiety appended to the CD cavity does not have enough motion to be self-included into the same CD.

As reported in a previous work, the excited-state dynamics of TPT in water at pH 6.24 is characterized by bi- or tri-exponential fluorescence decays, depending on the excitation wavelength (371 or 433 nm), as three different ground-state populations, i.e., E, C, and Z, co-exist under these experimental conditions [91]. The emission lifetimes are: *τ*_E*_ = 42 ps, *τ*_C*_ = 0.63 ns, and *τ*_Z*_ = 5.80 ns. Irreversible excited-state inter- or intramolecular PT (ESiPT or ESIPT) reactions occur with time constants spanning from the fs to ps time domains. The ESiPT reactions refer to: (1) the fast deprotonation (*τ*_ESiPT-oE*1_ = 42 ps) of the directly excited open E at the 10-hydroxyl group to generate A* which relaxes to S_0_ with a lifetime of 0.41 ns (observed at pH = 12.15); and (2) the slow deprotonation (*τ*_ESiPT-C*_ = 680 ps) of C*, directly excited or also coming from an ultrafast protonation (*τ*_ESiPT-oE*2_ < 10 ps) of open E* to give Z*. The ESIPT reaction concerns the ultrafast (*τ*_ESIPT-cE*_ < 10 ps) formation of Z* occurring from a directly excited closed E.

**Figure 5:**
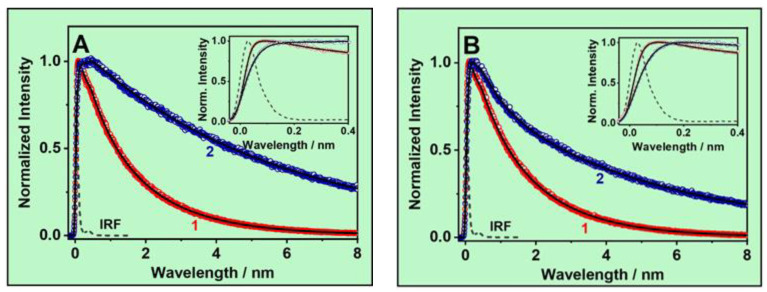
Normalized (to the maximum of intensity) ps-emission decays of (1) RB-RM-βCD and (2) TPT:RB-RM-βCD ([guest]/[host], where guest = TPT and host = RB-RM-βCD, ~4) in water solutions (pH ~6.2). The excitation is at 371 nm, while the gating wavelengths are (**A**) 540/565 and (**B**) 670 nm. The solid lines are from the best fit of the experimental data. IRF is the instrumental response function. Each inset shows a zoom of the dynamics at shorter times (≤1 ps).

To shed more light on the photobehavior of the TPT:RB-MeβCD complex, fluorescent lifetime experiments were performed, exciting at 371 nm (where mainly caged E absorbs) and interrogating over the whole range of emission spectra. Figure 5 shows the normalized emission decays of excited (1) RB-RM-βCD 1.1 × 10^−5^ M and (2) TPT:RB-RM-βCD ([guest]/[host] ~4) in water solutions at pH ~6.2. The observation wavelengths are: (A) 540/565 and (B) 670 nm (more details are given in Figure S8).

Table 2 gathers the corresponding fitting decay parameters *τ*_i_, *a*_i_, and *c*_i_ obtained from the multi-exponential fit of the emission decays of TPT:RB-RM-βCD in water solutions (pH ~6.2) at three different [guest]/[host] values upon excitation at 371 nm. Additional observation wavelengths and [guest]/[host] ratios are given in Table S3.

It is worth recalling that the observed photodynamics represents a global behavior of free and complexed TPT structures. In the presence of RB-RM-βCD, the fluorescence decays fit to a tri-or four-exponential model if the analyzed region is in the green (500 nm) or in the yellow/red (540–670 nm), respectively. The emission lifetimes from the best fit are *τ*_1_ = 39–40 ps, *τ*_2_ = 580–590 ps, *τ*_3_ = 1.7, and *τ*_4_ = 5.6–5.7 ns. They preserve their own values among the investigated [guest]/[host] ratios (0.38–4.16). *τ*_1_-component decays at 500 nm with very low contributions (1% at all the [guest]/[host] ratios), while it rises at lower energies (540–670 nm). The other components, *τ*_2_, *τ*_3_, and *τ*_4_, decay at all the gated wavelengths, with maxima contributions for all the [guest]/[host] values at 670 (4–6%), 580 (7–47%), and 540 (97–100%) nm, respectively. The reduction in the *c*_3_ value with the [guest]/[host] ratio at 580 nm was due to the simultaneous growth of *c*_4_ (from 49 to 92%) at this wavelength. The *τ*_1_ and *τ*_4_ values are fairly similar to those found for TPT:DM-βCD (38 ps and 5.66 ns) and TPT:TM-βCD (39 ps and 5.67 ns) complexes [30]. Hence, we assign them to a combination of free and caged E* and Z* structures. Furthermore, since *τ*_1_ is decaying in the green region and rising in the yellow/red part, it reflects the occurrence of an excited-state process in the TPT:RB-RM-βCD complex. One reasonable process could be, in agreement with our earlier results, an ESiPT involving a caged open E* to give the corresponding A*. The emissions of a caged A* of 810 and 440 ps were observed for the TPT:DM-βCD and TPT:TM-βCD systems, respectively. For TPT:RB-RM-βCD, it may well correspond to *τ*_2_ (580–590 ps), although it should be pointed out that this lifetime comprises the time constant of other species displaying similar behavior: (1) the RB:RM-βCD complex (*τ*_RB:RM-βCD_ = 585 ps) and (2) the free form of C* (*τ*_C*_ = 630 ps for TPT in water at pH 6.24 [91]). The existence of species (2) will be confirmed in the text below. Another excited-state process competing with the ESiPT could be a FRET between the open E* and RB, whose possibility due to the large spectral overlap between the emission of TPT and the absorption of RB (as shown in Figure S3) was discussed in the preceding section. 

**Figure 6:**
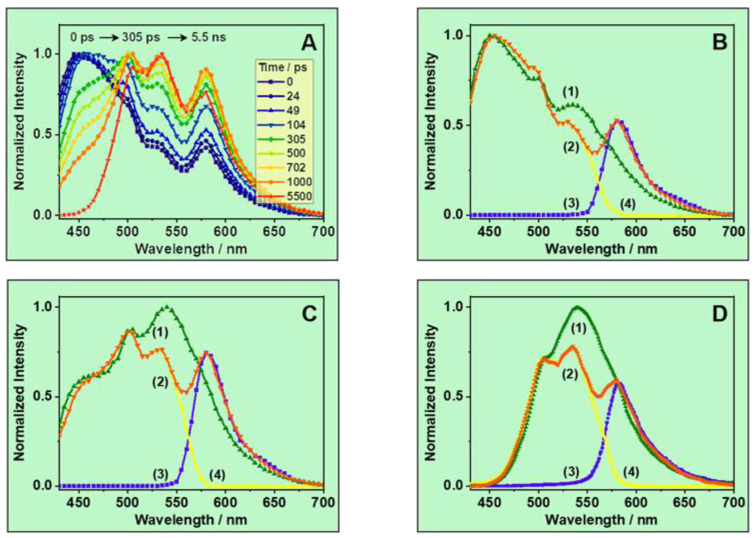
(**A**) Normalized time-resolved emission spectra (TRES) of TPT:RB-RM-βCD ([guest]/[host] ~4) in a water solution (pH ~6.2). (**B**–**D**) Comparison between the TRES of TPT (1); TPT:RB-RM-βCD ([guest]/[host] ~4) (2); and RB-RM-βCD (3) in water solutions (pH ~6.2) gating at a delay time of (**B**) ~50 ps, (**C**) 500 ps, and (**D**) ~5 ns. Spectrum (4) is the difference between spectra (3) and (1). The excitation wavelength was at 371 nm.

To further confirm the existence of a FRET process between TPT and RB, we recorded the TRES of TPT:RB-RM-βCD in water upon excitation at 371 nm (Figure 6A and Figure S9).

**Scheme 3:**
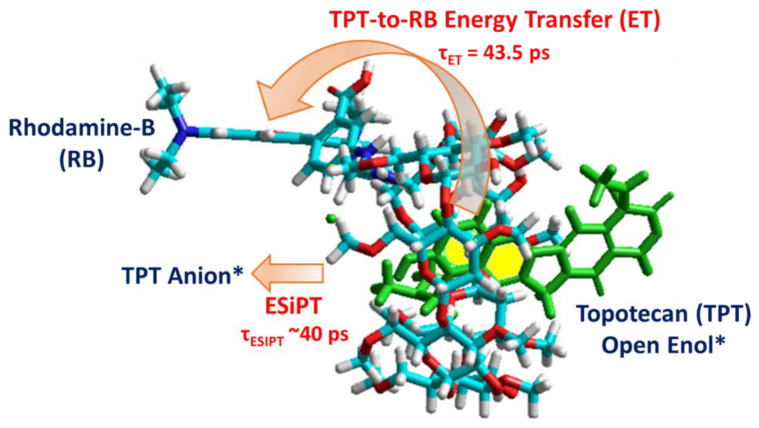
Proposed molecular structure of the complex TPT:RB-ME-βCD and the two competitive excited-state processes occurring in caged TPT*: excited-state intermolecular proton transfer (ESiPT) in TPT and energy transfer (ET) from the TPT E* to RB moiety. The stars for the enol and anion forms indicate an electronically excited state of these species.

The analysis of the spectral evolution at different delay times reveals the presence of fast (sub-ns regime) and slower (ns time regime) processes in the excited species involved. We can divide the TRES behavior into two parts: the 430–500 nm part, where the emission is from free/caged E* and free C* forms, and another 500–700 nm part, where the emission mainly originates from the free/caged Z* and RB*. The behavior of TRES agrees with the assignments made using the fluorescent lifetime measurements. A fast growth (within the ps laser pulse) of the signal from caged Z* ($\lambda_{em}^{max}$~540 nm) suggests, as in previous results, a fast sub-ps (<10 ps) Z* formation from a closed, more reactive E* form. The direct excitation of the caged Z* cannot be excluded under these conditions. Figure 6B,D show a comparison of the TRES of TPT, TPT:RB-RM-βCD ([TPT]/[RB-RM-βCD]~4), and RB-RM-βCD in water solutions (pH~6.2) gating at a delay time of (B) ~50 ps, (C) 500 ps, and (D) ~5 ns. It can be observed that the emission band related to A* ($\lambda_{em}^{max}$= 535 nm, Figure 6B) has a reduced intensity compared to the free TPT, thus suggesting that the excited-state formation of A* (photoproduced after the deprotonation of the caged open E*) is competing with an additional process which we assign to a FRET between the caged open E* and RB. Therefore, the shorter lifetime, *τ*_1_ = ~40 ps, should correspond to a combination of both the ESiPT and FRET events. Scheme 3 shows the two competitive excited-state processes, ESiPT and FRET, observed for the excited TPT:RB-RM-βCD complex.

Application of the Förster Theory for Non-Radiative FRET. In this section, we apply the FRET method to our system in order to determine the ET efficiency between the caged TPT and RB bonded to the CD cage. Using the emission spectrum of TPT:DM-βCD and its Φ_F_ value (0.20), we estimated an R_0_ value of 44 Å. The used [TPT]_0_ was 5.60 μM. The observed and corrected (E_Obs(c)_) efficiencies for the ET process involving TPT (5.60 μM) and RB at different concentrations of RB-RM-βCD are shown in Table S4. We obtained an E_Obs(c)_ (average) of 40%, which allowed to calculate an r value of 45 Å. The estimated k_ET_ was 2.3 × 10^10^ s^−1^ (calculated using the shortest lifetime of TPT:DM-βCD, *τ*_D_ = 38 ps) and the *τ*_ET_ = 43.5 ps.

**Figure 7:**
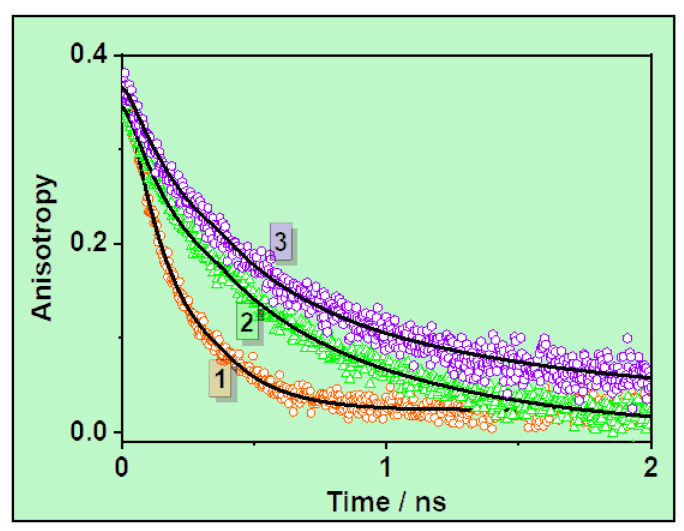
Anisotropy decays of (1) RB, (2) RB-RM-βCD, and (3) TPT:RB-RM-βCD in PBS solutions at pH 7.41, exciting at 510 and observing at 580 nm.

Time-resolved anisotropy measurements. To explore the robustness of the TPT:RB-RM-βCD complex, we also carried out time-resolved emission anisotropy experiments. Figure 7 shows emission anisotropy r(t) decays of RB, RB-RM-βCD, and TPT:RB-RM-βCD in PBS solutions at pH 7.41, exciting at 510 and observing at 580 nm. To begin with TPT, in water at pH = 6.24, we observed a rotational time (ϕ) of 156 ps. Based on the Stokes–Einstein–Debye hydrodynamic theory, we found that the experimental value is quite similar to the theoretical one (174 ps) obtained by modeling the molecule as a prolate ellipsoid rotor under stick-boundary conditions [91]. This indicates that strong H-bonding interactions between TPT and the surrounding water molecules affect its rotational relaxation time. The anisotropy decay of RB is mono-exponential (ϕ = 172 ps) whereas those of RB-RM-βCD and TPT:RB-RM-βCD are bi-exponential. The shorter time, ϕ_1_, is 219 ps in both cases. On the other side, ϕ_2_, the longer component, is 859 ps for RB-RM-βCD and 1.28 ns for the TPT:RB-RM-βCD complex. The increase in ϕ_2_ reflects the complex formation and its robustness. Applying the hydrodynamic theory (Table S5), we found that the rotational times calculated under stick-boundary conditions (*τ*_stick_ = 1100 and 1910 ps for RB-RM-βCD and TPT:RB-RM-βCD, respectively) together with the theoretical volumes (V_theor_ = 3783 and 5145 Å^3^ for RB-RM-βCD and TPT:RB-RM-βCD, respectively) are not so far from the experimental ones (ϕ_2_ = 859 and 1280 ps for RB-RM-βCD and TPT:RB-RM-βCD, respectively; V_exp_ = 3473 and 5176 Å^3^ for RB-RM-βCD and TPT:RB-RM-βCD, respectively).

Nowadays, the ability of modified CDs to cross or interact with biological barriers is the subject of strong investigation [96,97,98]. In particular, a direct spectroscopic detection of CDs in biological environments is a challenging task, as native CDs show no UV–vis light absorption and therefore no emission. More than 30 years ago, the first fluorophore appended CD detectable by fluorescent imaging techniques was reported [99]. Moreover, with the increased interest in CD-based drug delivery nanosystems, fluorescent CDs also gained importance from this perspective [100,101,102]. By combining a luminescent CD with a well-known antitumoral drug having the optimal requisites to undergo an ET process, this will create a luminescent supramolecular complex allowing a direct display of the drug release within the biological tissues, as has been very recently reported [103,104,105].

## 4. Conclusions

The findings reported and discussed in this work deal with the dynamics of the anticancer drug TPT in aqueous solutions (pH~6.2) in the presence of a rhodamine-labeled methylated βCD (RB-RM-βCD). The most stable TPT structure inside the CD pocket is the E one. A stable and robust TPT:RB-RM-βCD 1:1 complex is produced with a K_eq_ value of ~4 × 10^4^ M^−1^, which is comparable to those obtained for the interaction of TPT with DM-βCD (2.4 ± 0.5 × 10^4^ M^−1^) and TM-βCD (3.7 ± 0.8 × 10^4^ M^−1^). The emission intensity of an encapsulated TPT is clearly reduced in the presence of the hosting system due to the synergic effect of the CD restriction and an ET process occurring between the confined drug and the RB-labeled CD. The fluorescence decays recorded for the TPT:RB-RM-βCD complex fit to a multi-exponential model with emission lifetimes of: *τ*_1_ = 39–40 ps, *τ*_2_ = 580–590 ps, *τ*_3_ = 1.7, and *τ*_4_ = 5.6–5.7 ns. *τ*_1_ and *τ*_4_ are assigned to a combination of free and caged open E* and Z* structures, respectively. The emission of a caged A* may well correspond to τ_2_ (580–590 ps), although this time constant is a mixture of more than one species (A*, RB:RM-βCD complex, and C*). It is evidenced from TRES that the A* emission band shows less intensity compared to the case of free TPT, thus strengthening the occurrence of an ET between the caged open E* and RB. The FRET experiments and analysis give a TPT-to-RB ET efficiency of 40%. The anisotropy decay of a free RB is mono-exponential with a rotational time of 172 ps, whereas those of RB-RM-βCD and TPT:RB-RM-βCD are bi-exponential. In these cases, we observed the same shorter component (219 ps), while the longer one grows from 859 ps to 1.18 ns for RB:RM-βCD and TPT:RB-RM-βCD, respectively. This is further evidence of the complex formation and its robustness. These results may help in the design of new emissive CD-based host–guest nanoarchitectures displaying an efficient ET, improving their use in fluorescence techniques for drug delivery monitoring.

## Figures and Tables

**Table 1:** Time constants (*τ*_i_), normalized (to 100) pre-exponential factors (*a*_i_), and contributions (*c*_i_) obtained from the multi-exponential fit of the emission decays of RB 2.9 µM in water solutions (pH ~6.2), both without and after the addition of increasing amounts (from 0.2 to 20 mM) of DM-βCD. The excitation and observation wavelengths are 371 and 580 nm, respectively.

(DM-βCD) /mM	*τ*_1_/ps ± 50	*a*_1_ /%	*c*_1_ /%	*τ*_2_/ns ± 0.20	*a*_2_ /%	*c*_2_ /%	*τ*_3_/ns ± 0.30	*a*_3_ /%	*c*_3_ /%
0				1.67	100	100			
0.2	560	16	6	1.67	80	85	3.50	4	9
0.3	590	21	8	1.67	76	84	3.60	3	8
0.5	600	24	10	1.67	73	82	3.90	3	8
0.8	590	28	12	1.67	68	78	3.80	4	10
3	580	42	17	1.67	47	57	3.40	11	26
7	590	42	17	1.67	44	50	3.40	14	33
13	580	40	15	1.67	42	45	3.30	18	40
20	580	36	12	1.67	39	39	3.30	25	49

**Table 2:** Time constants (*τ*_i_), normalized (to 100) pre-exponential factors (*a*_i_), and contributions (*c*_i_) obtained from the multi-exponential fit of the emission decays of TPT:RB-RM-βCD in water solutions (pH ~6.2) at three different [guest]/[host] values upon the excitation at 371 nm, as observed in the table. The negative signs for *a*_1_ and *c*_1_ indicate a rising component in the emission signal.

**λ_obs_ = 500 nm**												
$\frac{\left[guest\right]}{\left[host\right]}$	***τ*_1_/ps** **± 15**	***a*_1_** **/%**	***c*_1_** **/%**	***τ*_2_/ps** **± 50**	***a*_2_** **/%**	***c*_2_** **/%**	***τ*_3_/ns** **± 0.2**	***a*_3_** **/%**	***c*_3_** **/%**	***τ*_4_/ns** **± 0.3**	***a*_4_** **/%**	***c*_4_** **/%**
0.38	39	52	1	590	8	2	-	-	-	5.7	40	97
1.14	40	43	1	590	11	2	-	-	-	5.6	46	97
4.16	40	39	1	580	15	3	-	-	-	5.6	46	96
**λ_obs_ = 670 nm**												
$\frac{\left[guest\right]}{\left[host\right]}$	***τ*_1_/ps** **± 15**	***a*_1_** **/%**	***c*_1_** **/%**	***τ*_2_/ps** **± 50**	***a*_2_** **/%**	***c*_2_** **/%**	***τ*_3_/ns** **± 0.2**	***a*_3_** **/%**	***c*_3_** **/%**	***τ*_4_/ns** **± 0.3**	***a*_4_** **/%**	***c*_4_** **/%**
0.38	39	−100	−100	590	25	6	1.7	53	38	5.7	22	56
1.14	40	−100	−100	590	27	5	1.7	32	18	5.6	41	77
4.16	40	−100	−100	580	25	4	1.7	13	5	5.6	62	91

## Data Availability

Not applicable.

## Supplementary Materials

The following supporting information can be downloaded at: https://www.mdpi.com/article/10.3390/pharmaceutics15061620/s1. Scheme S1. Illustration of the ground-state equilibrium between the lactone and carboxylate forms of TPT in a water solution. Scheme S2. Illustration of the TPT species in water at pH 6.24. Figure S1. Normalized absorption spectra of TPT:RB-RM-βCD, TPT:DM-βCD), and TPT:TM-βCD in water at pH ~6.2 or in phosphate buffered saline (PBS) solutions at pH = 7.23 (2,3). Figure S2. Normalized absorption spectra of TPTand RB-RM-βCD in water solutions (pH ~6.2). Figure S3. Normalized absorption and emission spectra of TPT and RB-RM-βCD in water solutions (pH ~6.2). Figure S4. Normalized emission spectra of RB 2.9 µM in water solutions (pH ~6.2) without and after addition of DM-βCD of different concentrations. Figure S5. Absorbance variation of RB in water at pH ~6.2 with DM-βCD concentration observed at 554 nm. Figure S6. (A) Normalized ps-emission decays of RB 2.9 µM in water solutions (pH ~6.2) without and after addition of DM-βCD at different concentrations. (B) Dependence of the *c*1-to-*c*3 ratio (*c*1/*c*3) with DM-βCD concentration, where *c*1 and *c*3 are the contributions of *τ*1 and *τ*3 components in the emission decays at 630 nm. Figure S7. Normalized ps-emission decays of RB-RM-βCD in PBS solutions (pH = 7.3) at different concentrations. Figure S8. Normalized ps-emission decays of TPT:RB-RM-βCD in water solutions (pH ~6.2) at five different [guest]/[host] ratios. Figure S9. TRES of TPT:RB-RM-βCD in a water solution (pH ~6.2) upon excitation at 371 nm and with a [TPT]/[RB-RM-βCD] value of ~4. Table S1. Time constants, normalized pre-exponential factors and contributions obtained from the multi-exponential fit of the emission decays of RB 2.9 µM in water solutions (pH ~6.2) without and after addition of increasing amounts (from 0.2 to 20 mM) of DM-βCD. Table S2. Time constants, normalized pre-exponential factors and contributions obtained from the multi-exponential fit of the emission decays of RB-RM-βCD in PBS solutions (pH = 7.3) at four different concentrations of RB-RM-βCD. Table S3. Time constants, normalized pre-exponential factors and contributions obtained from the multi-exponential fit of the emission decays of TPT:RB-RM-βCD in water solutions (pH ~6.2) at five different [guest]/[host] ratios. Table S4. Observed and corrected (EObs(c)) efficiencies for the ET process involving TPT (5.60 μM) and RB at different concentrations of RB-RM-βCD. Table S5. Rotational relaxation times (φ) and molecular volumes (Vexp) of RB, RB-RM-βCD, and TPT:RB-RM-βCD in PBS solutions at pH 7.41. Description of BH Model using absorption Data.

## Abbreviations

TPT	topotecan
RB-RM-βCD	6-deoxy-6-[(5/6)-rhodaminylthioureido]-randomly methylated-βCD
FRET	Förster resonance energy transfer
CPT	camptothecin
Top1	topoisomerase I
RNA	ribonucleic acid
DNA	deoxyribonucleic acid
RB	rhodamine B
CD	cyclodextrin
HP-βCD	2-hydroxypropyl-βCD
SBE-βCD	sulfobutylether-βCD
RM-βCD	randomly methylated βCD
CT	charge transfer
PT	proton transfer
ET	energy transfer
ELISA	Enzyme-Linked ImmunoSorbent Assay
DM-βCD	heptakis(2,6-di-O-methyl)-βCD
TM-βCD	heptakis(2,3,6-tri-O-methyl)-βCD
^1^HNMR	proton nuclear magnetic resonance
Me	methyl
UV	ultraviolet
TCSPC	time-correlated single-photon counting
IRF	instrumental response function
E	enol
C	cation
Z	zwitterion
iHBs	intermolecular H-bonds
A	anion
PBS	phosphate-buffered saline
TRES	time-resolved emission spectra
D	donor
A	acceptor
BH	Benesi–Hildebrand
H1	heptakis-[6-deoxy-6-(3-sulfanylpropanoic acid)]-βCD
H2	heptakis-[6-deoxy-6-(2-sulfanylacetic acid)]-βCD
ESiPT	excited-state intermolecular PT
ESIPT	excited-state intramolecular PT
