# Supplementary material for: Robust Inclusion Complex of Topotecan Comprised within a Rhodamine-Labeled β-Cyclodextrin: Competing Proton and Energy Transfer Processes
Source: Pharmaceutics. 2023 May 30;15(6):1620. doi: 10.3390/pharmaceutics15061620 (PMC10303446; doi:10.3390/pharmaceutics15061620)
Supplement: Supplementary file 1 [file pharmaceutics-15-01620-s001.zip › pharmaceutics-2348745-supplementary.pdf]

Supplementary Materials: Robust Inclusion Complex of Topotecan Comprised within a Rhodamine-Labeled β-Cyclodextrin: Competing Protonand Energy Transfer Processes

Maria Rosaria di Nunzio and Abderrazzak Douhal

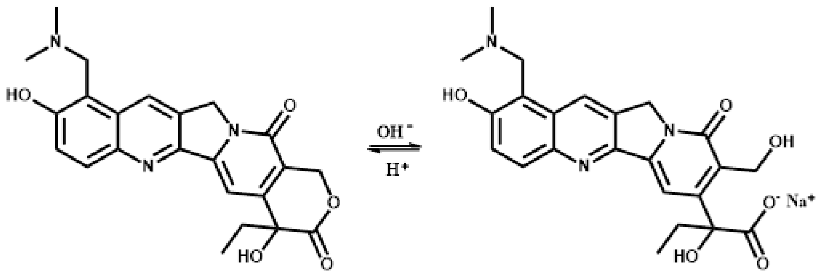

Scheme S1. Illustration of the ground-state equilibrium between the lactone and carboxylate forms of TPT in a water solution.

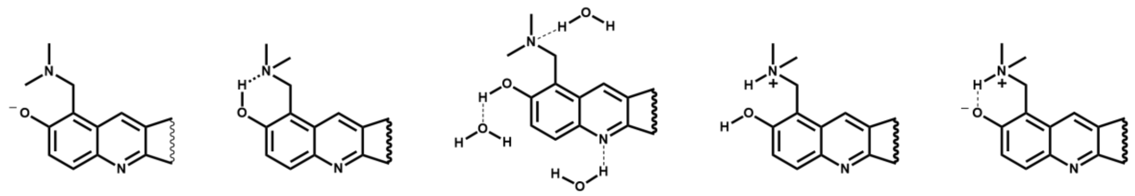

Anion (A) Closed Enol (closed E) Open Enol (open E) Cation (C) Zwitterion (Z)

Scheme S2. Illustration of the TPT species in water at pH 6.24: anion (A), enol (closed and open E), cation (C), and zwitterion (Z) species. For simplicity, only the quinoline moieties of the structures are shown.

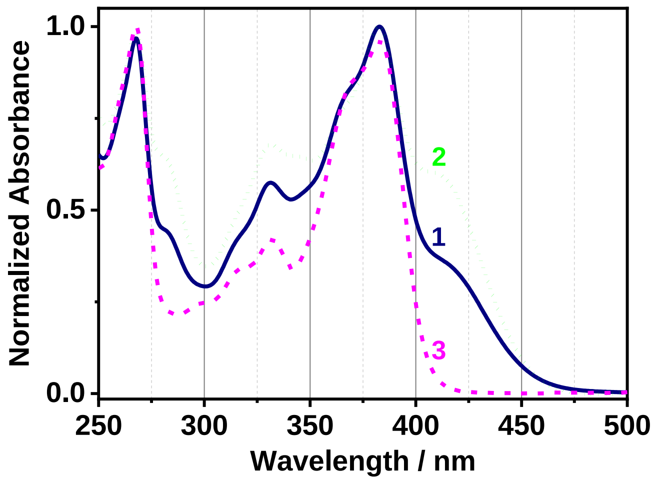

Figure S1. Normalized (to the maximum of intensity) absorption spectra of TPT:RB-RM-βCD (1, solid line), TPT:DM-βCD (2, dotted line), and TPT:TM-βCD (3, dashed line) in water at pH ~6.2 (1) or in phosphate-buffered saline (PBS) solutions at pH = 7.23 (2,3).

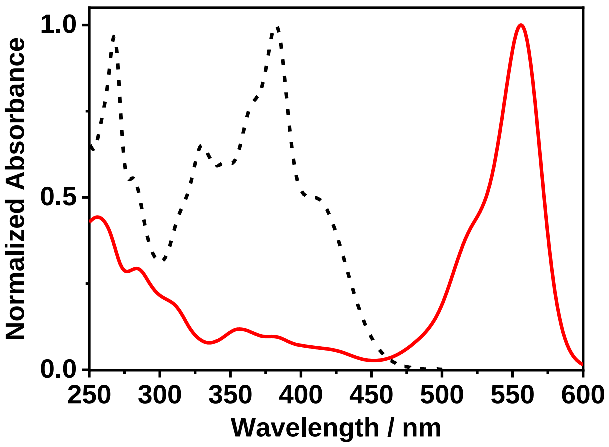

Figure S2. Normalized (to the maximum of intensity, i.e., 382 and 556 nm for TPT and RB-RM-βCD, respectively) absorption spectra of TPT (black dashed line) and RB-RM-βCD (red solid line) in water solutions (pH ~6.2).

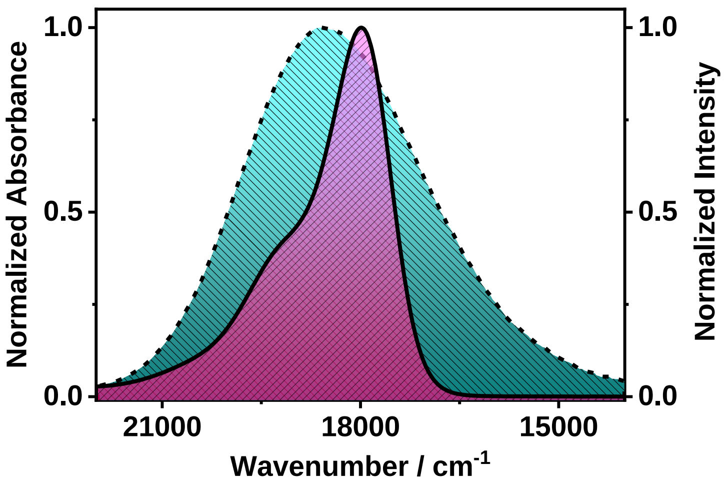

Figure S3. Normalized (to the maximum of intensity) absorption (solid line) and emission (dashed line) spectra of TPT and RB-RM-βCD, respectively, in water solutions (pH ~6.2).

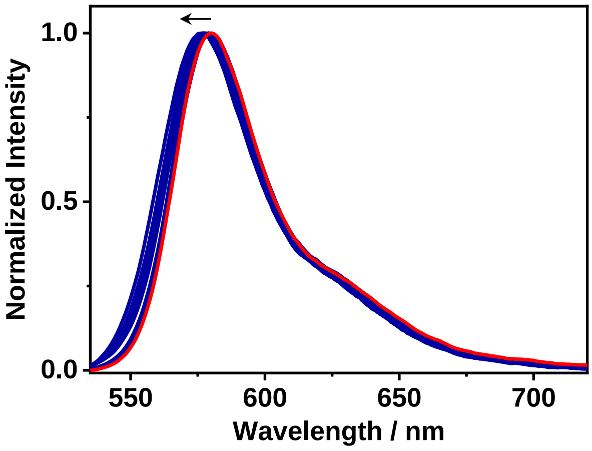

Figure S4. Normalized emission spectra of RB 2.9 μM in water solutions (pH ~6.2) without (red line) and after (blue lines) the addition of DM-βCD in different concentrations (from 0.2 to 20 mM). The blue-shifted spectra are corrected for the dilution effect.

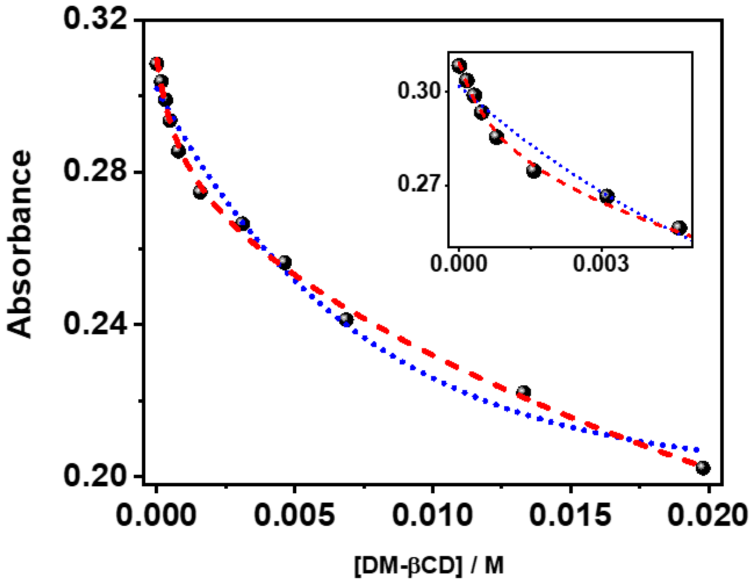

Figure S5. Absorbance variation of RB in water at pH ~6.2 with DM-βCD concentration observed at 554 nm. The red dashed line is from the best fit assuming the formation of 1:1 and 1:2 complexes, while the blue dotted one is from the best fit only assuming the formation of the 1:1 complex. Insert: the zoom of Figure S4 at low DM-βCD concentrations.

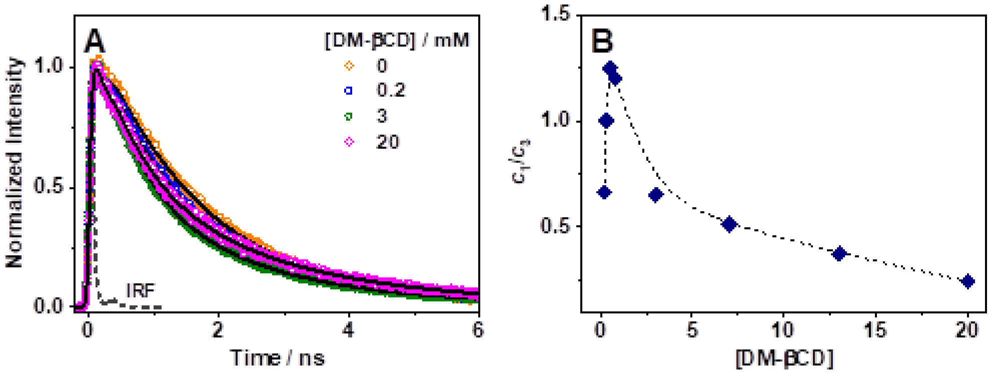

Figure S6. (A) Normalized (to the maximum of intensity) ps-emission decays of RB 2.9 μM in water solutions (pH ~6.2) without and after the addition of DM-βCD at different concentrations (0.2, 3, and 20 mM). The excitation and observation wavelengths were 371 and 630 nm, respectively. The solid lines are from the best fit of the experimental data. IRF is the instrumental response function. (B) Dependence of the c_1-to-c_3 ratio (c_1/c_3) with DM-βCD concentration, where c_1 and c_3 are the contributions of τ_1 and τ_3 components in the emission decays at 630 nm. The dashed line in (B) only serves to guide the eyes.

Table S1. Time constants (τ_i), normalized (to 100) pre-exponential factors (a_i), and contributions (c_i) obtained from the multi-exponential fit of the emission decays of RB 2.9 μM in water solutions (pH ~6.2) without and after the addition of increasing amounts (from 0.2 to 20 mM) of DM-βCD. The excitation was at 371 nm and the observation wavelengths are indicated in the table.

[DM-βCD] /mM	λ_{obs} /nm	τ_1/ps ± 50	a_1 /%	c_1 /%	τ_2/ns ± 0.20	a_2 /%	c_2 /%	τ_3/ns ± 0.30	a_3 /%	c_3 /%
0	560				1.67	100	100			

	580					100	100			
	630					100	100			
0.2	560	560	20	7	1.67	76	84	3.50	4	9
	580		16	6		80	85		4	9
	630		19	7		77	85		4	8
0.3	560	590	27	11	1.67	70	82	3.60	3	7
	580		21	8		76	84		3	8
	630		25	10		72	82		3	8
0.5	560	600	31	13	1.67	67	80	3.90	2	7
	580		24	10		73	82		3	8
	630		27	11		70	81		3	8
0.8	560	590	35	15	1.67	62	76	3.80	3	9
	580		28	12		68	78		4	10
	630		32	13		64	77		4	10
3	560	580	46	20	1.67	43	52	3.40	11	28
	580		42	17		47	57		11	26
	630		46	20		45	56		9	24
7	560	590	45	18	1.67	39	45	3.40	16	37
	580		42	17		44	50		14	33
	630		47	20		41	50		12	30
13	560	580	41	15	1.67	37	38	3.30	22	47
	580		40	15		42	45		18	40
	630		45	18		39	45		16	37
20	560	580	35	11	1.67	34	31	3.30	31	58
	580		36	12		39	39		25	49
	630		40	15		38	40		22	45

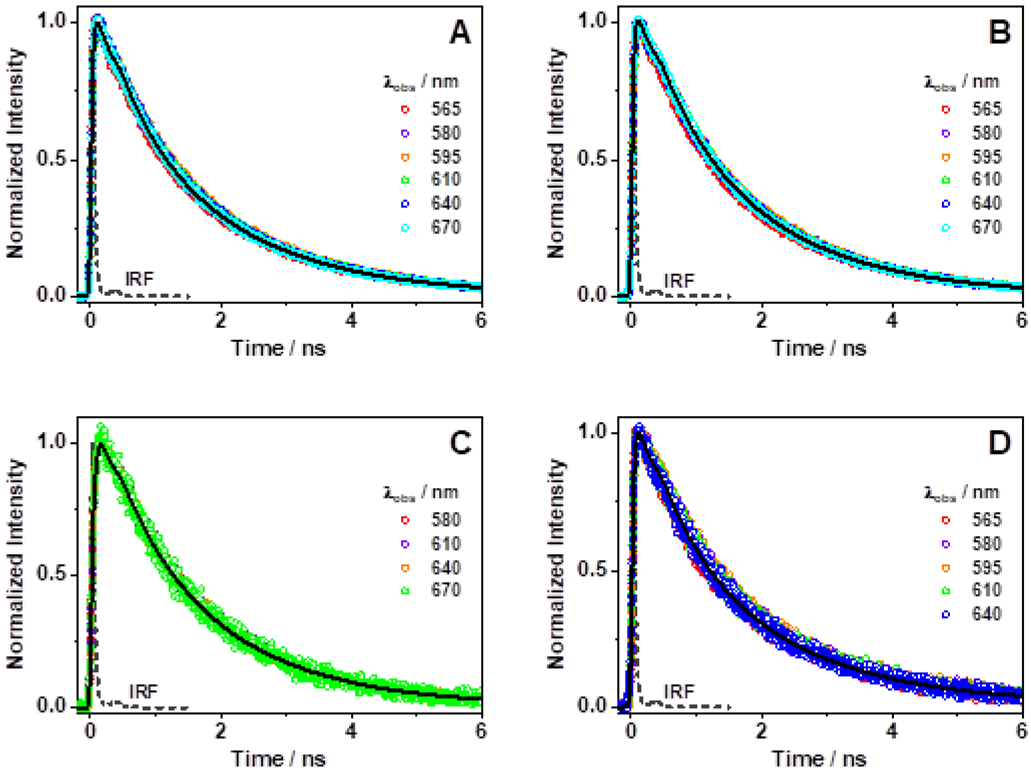

Figure S7. Normalized (to the maximum of intensity) ps-emission decays of RB-RM-βCD in PBS solutions (pH = 7.3) at different concentrations: (A) 1.1 × 10^{-4} M, (B) 1.1 × 10^{-5} M, (C) 1.1 × 10^{-6} M, and (D) 1.1 × 10^{-7} M. The excitation wavelength is at 371 nm, while the observation ranges are indicated in the inset. The solid lines are from the best fit of the experimental data. IRF is the instrumental response function.

Table S2. Time constants (τ_i), normalized (to 100) pre-exponential factors (a_i), and contributions (c_i) obtained from the multi-exponential fit of the emission decays of RB-RM-βCD in PBS solutions (pH = 7.3) at four different concentrations of RB-RM-βCD upon excitation at 371 nm and observation as indicated in the table.

[RB-RM-βCD] /10^{-7} M	λ_{obs} /nm	τ_1/ps ± 50	a_1 /%	c_1 /%	τ_2/ns ± 0.2	a_2 /%	c_2 /%	τ_3/ns ± 0.3	a_3 /%	c_3 /%
1.1	565	580	15	10	1.6	79	78	3.4	6	12
	580		17	7		78	82		5	11
	595		17	7		78	82		5	11
	610		20	9		75	80		5	11
	640		22	10		73	79		5	11
11	580	580	16	6	1.6	79	83	3.4	5	11
	610		18	7		77	82		5	11
	640		18	7		77	82		5	11
	670		20	8		75	80		5	12
110	565	590	26	11	1.6	68	77	3.3	6	12
	580		19	8		76	82		5	10
	595		18	7		77	82		5	11
	610		21	8		74	81		5	11
	640		21	8		74	82		5	10
	670		22	9		73	81		5	10
1100	565	590	29	10	1.7	66	78	3.5	5	12
	580		24	8		71	81		5	11
	595		23	8		72	81		5	11
	610		25	8		71	81		4	11
	640		26	9		70	81		4	10
	670		27	10		69	80		4	10

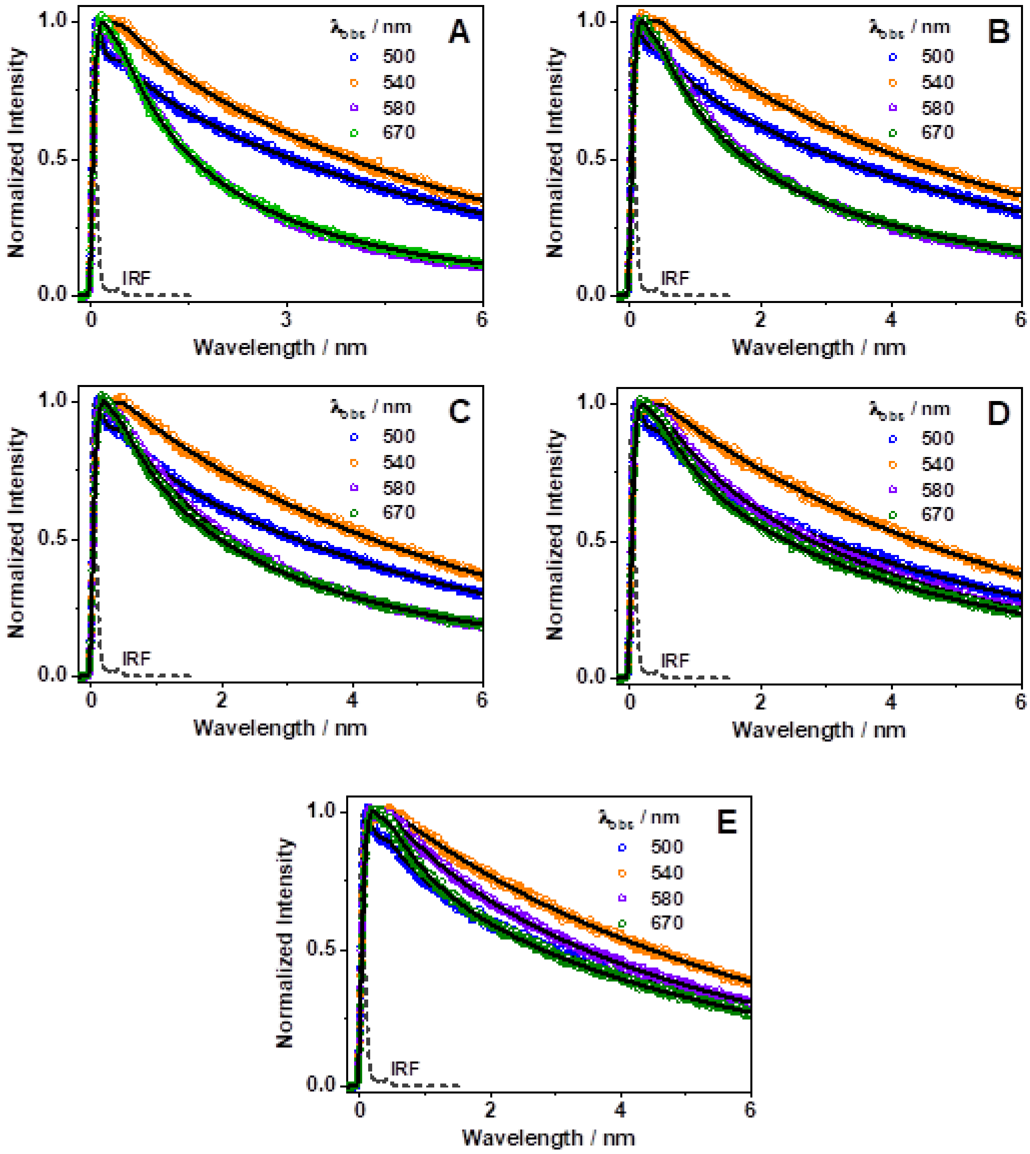

Figure S8. Normalized (to the maximum of intensity) ps-emission decays of TPT:RB-RM-βCD in water solutions (pH ~6.2) at five different [guest]/[host] ratios (guest = TPT; host = RB-RM-βCD): (A) 0.38, (B) 0.76, (C) 1.14, (D) 2.27, and (E) 4.16. The excitation wavelength was at 371 nm, while the observation wavelengths are indicated in the inset. The solid lines are from the best fit of the experimental data. IRF is the instrumental response function.

Table S3. Time constants (τ_i), normalized (to 100) pre-exponential factors (a_i), and contributions (c_i) obtained from the multi-exponential fit of the emission decays of TPT:RB-RM-βCD in water solutions (pH ~6.2) at five different [guest]/[host] ratios upon excitation at 371 nm and observation as indicated in the table. The negative signs for a_1 and c_1 indicate a rising component in the emission signal.

λ_{obs} = 500 nm												
$\frac{[guest]}{[host]}$	τ_1/ps ± 15	a_1 /%	c_1 /%	τ_2/ps ± 50	a_2 /%	c_2 /%	τ_3/ns ± 0.2	a_3 /%	c_3 /%	τ_4/ns ± 0.3	a_4 /%	c_4 /%
0.38	39	52	1	590	8	2	-	-	-	5.7	40	97
0.76	39	44	1	590	11	2	-	-	-	5.7	45	97
1.14	40	43	1	590	11	2	-	-	-	5.6	46	97
2.27	40	40	1	580	13	3	-	-	-	5.6	47	96
4.16	40	39	1	580	15	3	-	-	-	5.6	46	96
λ_{obs} = 540 nm												
$\frac{[guest]}{[host]}$	τ_1/ps ± 15	a_1 /%	c_1 /%	τ_2/ps ± 50	a_2 /%	c_2 /%	τ_3/ns ± 0.2	a_3 /%	c_3 /%	τ_4/ns ± 0.3	a_4 /%	c_4 /%
0.38	39	-100	-100	590	9	1	1.7	6	2	5.7	85	97
0.76	39	-100	-100	590	6	1	1.7	4	1	5.7	90	98
1.14	40	-100	-100	590	6	1	1.7	< 1	< 1	5.6	94	99
2.27	40	-100	-100	580	4	1	1.7	< 1	< 1	5.6	96	99
4.16	40	-100	-100	580	2	< 1	1.7	< 1	< 1	5.6	98	100
λ_{obs} = 580 nm												
$\frac{[guest]}{[host]}$	τ_1/ps ± 15	a_1 /%	c_1 /%	τ_2/ps ± 50	a_2 /%	c_2 /%	τ_3/ns ± 0.2	a_3 /%	c_3 /%	τ_4/ns ± 0.3	a_4 /%	c_4 /%
0.38	39	-100	-100	590	13	4	1.7	55	47	5.7	32	49
0.76	39	-100	-100	590	13	3	1.7	53	32	5.7	34	65
1.14	40	-100	-100	590	11	2	1.7	44	25	5.6	45	73
2.27	40	-100	-100	580	9	1	1.7	32	14	5.6	59	85
4.16	40	-100	-100	580	4	1	1.7	21	7	5.6	75	92
λ_{obs} = 670 nm												
$\frac{[guest]}{[host]}$	τ_1/ps ± 15	a_1 /%	c_1 /%	τ_2/ps ± 50	a_2 /%	c_2 /%	τ_3/ns ± 0.2	a_3 /%	c_3 /%	τ_4/ns ± 0.3	a_4 /%	c_4 /%
0.38	39	-100	-100	590	25	6	1.7	53	38	5.7	22	56
0.76	39	-100	-100	590	26	6	1.7	40	24	5.7	34	70
1.14	40	-100	-100	590	27	5	1.7	32	18	5.6	41	77
2.27	40	-100	-100	580	26	4	1.7	20	10	5.6	54	86
4.16	40	-100	-100	580	25	4	1.7	13	5	5.6	62	91

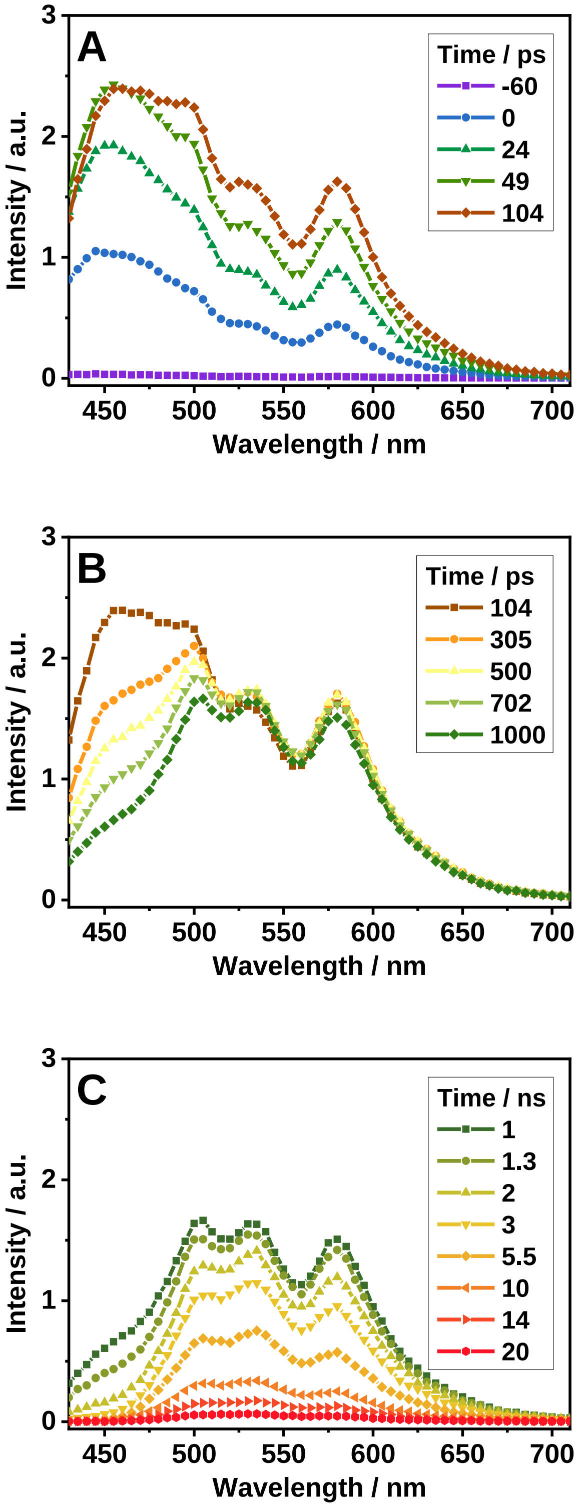

Figure S9. TRES of TPT:RB-RM-βCD in a water solution (pH ~6.2) upon ~~the~~ excitation at 371 nm and with a [TPT]/[RB-RM-βCD] value of ~4. The insets indicate the gating times of the TRES. (A): from – 60 to 104 ps, (B): from 104 to 1000 ps and (C): from 1 to 20 ns.

Table S4. Observed and corrected ($E_{Obs(c)}$) efficiencies for the ET process involving TPT (5.60 μM) and RB at different concentrations of RB-RM-βCD. f is the molar fraction of the 1:1 complex between TPT and RB-RM-βCD having an equilibrium constant $K_{eq} = 3.7 \times 10^4$ M^{-1} at 20 °C, and $E_{Obs(c)} = E_{Obs}/f$.

[RB-RM-βCD] / μM	f	$E_{Obs(c)}$ (%)
43	0.59	39
35	0.54	41
25	0.45	43
19	0.38	41
14.5	0.32	40
10.1	0.24	42
7.5	0.19	41
3.5	0.098	35

Table S5. Rotational relaxation times (φ) and molecular volumes (V_{exp}) of RB, RB-RM-βCD, and TPT:RB-RM-βCD in PBS solutions at pH 7.41 exciting at 510 and observing at 580 nm. The molecular systems were modeled as ellipsoid, non-hydrated rotors under both stick- and slip-boundary conditions using the Stokes–Einstein–Debye hydrodynamic theory. The calculated parameters are: τ_{stick}, τ_{slip} (rotational times), and V_{theor} (molecular volume).

System	A = C / Å	B / Å	Type of ellipsoid	V_{theor}[a] / Å^3	f[b]	τ_{stick} (τ_{slip}) / ps	φ / ps	V_{exp}[c] / Å^3
RB	9.8	14.8	Prolate	744	1.2	221 (23.4)	172	728
RB-RM-βCD	17	25	Prolate	3783	1.2	1100 (117)	219, 859	3473[d]
TPT:RB-RM-βCD	17	34	Prolate	5145	1.5	1910 (460)	219, 1280	5176[d]

[a] $V_{theor} = (4\pi a^2 b)/3$, where a = c and b hold for the semi-minor and the semi-major axes of the prolate ellipsoid, respectively. [b] The parameter f is a factor which takes into account the shape of the solute (f = 1 for a sphere, > 1 for non-spherical molecules). [c] $V_{exp} = (\varphi k_B T)/(\eta f C)$, where φ is the experimental rotational time, k_B is the Boltzmann constant (1.38×10^{-16} erg/K), T is the temperature (293 K), η is the viscosity of the medium (9.55×10^{-3} and 11×10^{-3} Poise for buffer and buffer in presence of CD, respectively), and C is a factor which is equal to 1 for stick-boundary conditions. [d] Calculated taking into account the longer φ.

Benesi–Hildebrand (BH) Model

BH Model from Absorption Data

When the cyclodextrin (CD) forms a 1:1 complex with the substrate (S), the binding constant (K_{eq}) can be expressed as:

$$K_{eq} = \frac{[S:CD]}{[CD]\cdot[S]} = \frac{X_i}{(C_{CD} - C_S X_i)(1 - X_i)} \quad (S1)$$

where [S:CD], [CD], and [S] are the equilibrium concentrations of the complex, free CD, and free S, respectively. C_{CD} and C_S are the concentrations of CD and S, while $X_i = [S:CD]/C_S$. Assuming that the molar extinction coefficients of the free and caged S at a certain wavelength are ε_0 and ε_∞, respectively (herein, the absorption of CD is usually equal to 0). Thus, when $C_{CD} = 0$, the absorption of the solution is $A_0 = \varepsilon_0 l C_S$, in which l is the length of the cell. If the analytical concentration of CD is C_{CD}^i, the apparent absorption of the solution is $A_i = \varepsilon_0 l C_S(1-X_i) + \varepsilon_\infty l C_S X_i$. If C_S is constant, then $A_i = A_0 + \Delta A X_i$, where $\Delta A = (\varepsilon_\infty - \varepsilon_0) l C_S$. If $C_{CD}^i \gg C_S$, the following equation can be obtained:

$$\frac{1}{K_{eq}} = \frac{(C_{CD}^i - C_S X_i)(1 - X_i)}{X_i} = C_{CD}^i \frac{(1 - X_i)}{X_i} = C_{CD}^i \frac{\Delta A}{(\Delta A_i - 1)} \quad (S2)$$

where $\Delta A_i = A_i - A_0 = \Delta A X_i$. Rearrangement of (2) gives:

$$\frac{1}{\Delta A_i} = \frac{1}{\Delta A} + \frac{1}{\Delta A K_{eq}} \cdot \frac{1}{C_{CD}^i} \quad (S3)$$

The double reciprocal plot of $1/\Delta A_i$ vs. $1/C_{CD}^{i}$ gives a slope of $1/\Delta A K_{eq}$ and an intercept of $1/\Delta A$. The intercept-to-slope ratio can be assumed to be the binding constant K_{eq}.

BH Model from Emission Data

In the case of a 1:1 stoichiometry, the relation between the fluorescence intensity variation and the CD concentration can be represented as:

$$\frac{1}{\Delta I_i} = \frac{1}{\Delta I_{max}} + \frac{1}{\Delta I_{max} K_{eq}} \cdot \frac{1}{C_{CD}^{i}} \quad (S4)$$

$$K_{eq} = \frac{1}{slope \cdot \Delta I_{max}} \quad (S5)$$

In (4), ΔI_i and ΔI_{max} refer to I_0 - I_i and $I_{max} - I_0$, in that order, with I_0, I, and I_{max} being the emission intensities of TPT in the absence of CD, at an intermediate CD concentration, and at a concentration of complete saturation, respectively, K_{eq} is the binding constant and C_{CD}^{i} is the CD concentration. From the plot of 1/ ΔI_i vs. $1/C_{CD}^{i}$, the value of K_{eq} has been determined from the intercept-to-slope ratio.

In both cases, the associated error of K_{eq}, dK_{eq}, is calculated as:

$$dK_{eq} = \left(\frac{dslope}{|slope|} + \frac{dintercept}{|intercept|}\right) \times |K_{eq}| \quad (S6)$$

Binding Constant for the Formation of 1:1 and 1:2 Complexes

In this case, we used the expression:

$$A = \frac{(A_0 + K_1 A_1[CD] + K_1 K_2 A_2 [CD]^2)}{(1 + K_1[CD] + K_1 K_2 [CD]^2)} \quad (S7)$$

where A is the absorbance of the system at different concentrations of CD, [CD] is the concentration of DM-βCD in the solution, A_0 is the absorbance of RB without DM-βCD, A_1 is the absorbance of RB forming 1:1 complex with DM-βCD (this parameter was left free during the fitting process), and A_2 is the absorbance of RB forming a 1:2 complex with DM-βCD.

Application of the Förster Theory for the Non-Radiative Energy Transfer

Resonant energy transfer is characterized by a rate constant (k_{DA}), which expresses the probability of the transfer:

$$k_{DA}(r) = \frac{1}{\tau_D}\left(\frac{R_0}{r}\right)^6 \quad (S8)$$

where τ_D is the donor fluorescence decay time. R_0 is the distance at which the probability of the energy transfer is equal to the probability of the internal deactivation of the excited state of the molecule, and r is the distance between donor and acceptor.

R_0 can be expressed as:

$$R_0 = 0.211 \times [\kappa^2 n^{-4} Q_D J(\lambda)]^{(1/6)} \quad (S9)$$

where κ^2 (the relative orientation in space of the transition dipoles of the donor and the acceptor) is assumed to be 2/3, n (the refractive index) is 1.333, and Q_D (the quantum yield of the donor in the absence of acceptor) is 0.20 (this value corresponds to the quantum yield of the TPT:DM-βCD complex). J(λ), the overlap integral, is given by:

$$J = \frac{\int_0^{\infty} F_D(\lambda)\varepsilon_A(\lambda)\lambda^4 d\lambda}{\int_0^{\infty} F_D(\lambda)\, d\lambda} \quad (S10)$$

where $F_D(\lambda)$ is the corrected fluorescence intensity of the donor in the wavelength range λ +$\Delta\lambda$, with the total intensity normalized to unity, and ε_A is the extinction coefficient of the acceptor at λ.

The transfer efficiency can be measured using the relative fluorescence intensity of the donor, in the absence (F_D) and presence (FDA) of the acceptor:

$$E = 1 - \frac{F_{DA}}{F_D} \quad (S11)$$

Using the following relationship:

$$E = \frac{R_0^6}{R_0^6 + r^6} \quad (S12)$$

We can calculate r combining Equations S11 and S12. The value of r can be finally used in Equation S8 to estimate the k_{DA}(r) value.
